# Supplementary material for: Id Proteins Promote a Cancer Stem Cell Phenotype in Mouse Models of Triple Negative Breast Cancer via Negative Regulation of Robo1
Source: Front Cell Dev Biol. 2020 Jul 17;8:552. doi: 10.3389/fcell.2020.00552 (PMC7380117; doi:10.3389/fcell.2020.00552)
Supplement: Supplementary file 1 [file Data_Sheet_1.PDF]

Supplementary Table S1. Top 25 up and down regulated genes identified in Id-depleted K1 cells ranked based on Q-value. Column 1 shows the gene symbols and annotation. Fold change in column 2 represents the ratio of gene expression change in Id1/3 knockdown versus control. Column 3 represents the Q-value for the difference between knockdown and control group. Column 4 shows the direction of change in gene expression.

Gene	Fold Change	Q-value	Direction
Mx2 :: myxovirus (influenza virus) resistance 2	26.6015	7.59E-08	UP
Oas1g :: 2'-5' oligoadenylatesynthetase 1G	14.9574	1.04E-07	UP
Oas3 :: 2'-5' oligoadenylatesynthetase 3	15.124	1.95E-07	UP
Cmpk2 :: cytidine monophosphate (UMP-CMP) kinase 2, mitochondrial	24.33	2.32E-07	UP
Stat1 :: signal transducer and activator of transcription 1	6.8186	3.29E-07	UP
Xaf1 :: XIAP associated factor 1	9.0697	4.06E-07	UP
Usp18 :: ubiquitin specific peptidase 18	30.489	4.10E-07	UP
Oas2 :: 2'-5' oligoadenylatesynthetase 2	36.1304	4.10E-07	UP
Ifit1 :: interferon-induced protein with tetratricopeptide repeats 1	18.5318	4.58E-07	UP
Gpr56 :: G protein-coupled receptor 56	21.8355	4.72E-07	UP
Zbp1 :: Z-DNA binding protein 1	13.582	4.72E-07	UP
Olfr65 :: olfactory receptor 65	8.3323	4.72E-07	UP
Parp14 :: poly (ADP-ribose) polymerase family, member 14	5.9386	5.22E-07	UP
Angptl4 :: angiopoietin-like 4	12.113	5.22E-07	UP
Irf7 :: interferon regulatory factor 7	13.4442	5.22E-07	UP
Gbp3 :: guanylate binding protein 3	10.1348	5.68E-07	UP
Stat2 :: signal transducer and activator of transcription 2	5.0425	6.80E-07	UP
Oasl2 :: 2'-5' oligoadenylatesynthetase-like 2	5.7617	7.46E-07	UP
Bst2 :: bone marrow stromal cell antigen 2	8.4297	7.46E-07	UP
Lypd3 :: Ly6/Plaur domain containing 3	4.2202	7.46E-07	UP
Iigp1 :: interferon inducible GTPase 1	14.3364	7.93E-07	UP
Pvrl1 :: poliovirus receptor-related 1	4.7014	7.93E-07	UP
Oas1b :: 2'-5' oligoadenylatesynthetase 1B	9.7557	8.90E-07	UP
Megf10 :: multiple EGF-like-domains 10	11.0025	1.04E-06	UP
Rtp4 :: receptor transporter protein 4	9.0028	1.04E-06	UP
H19 :: H19 fetal liver mRNA	2.8526	3.68E-06	DOWN
Mpzl2 :: myelin protein zero-like 2	3.1982	3.86E-06	DOWN
Cpox :: coproporphyrinogen oxidase	2.5431	4.32E-06	DOWN
Gpr116 :: G protein-coupled receptor 116	2.9257	5.34E-06	DOWN
Cep78 :: centrosomal protein 78	2.5606	8.09E-06	DOWN
Gpt2 :: glutamic pyruvate transaminase (alanine aminotransferase) 2	3.2592	8.29E-06	DOWN
Cth :: cystathionase (cystathionine gamma-lyase)	4.9146	1.01E-05	DOWN
Zfp420 :: zinc finger protein 420	2.6201	1.07E-05	DOWN

Gja1 :: gap junction protein, alpha 1	2.2993	1.73E-05	DOWN
Cldn9 :: claudin 9	4.2349	1.82E-05	DOWN
Rtkn2 :: rhotekin 2	3.3638	1.93E-05	DOWN
Aqp1 :: aquaporin 1	3.4829	2.80E-05	DOWN
Fermt1 :: fermitin family homolog 1 (Drosophila)	2.1462	3.05E-05	DOWN
Taf5 :: TAF5 RNA polymerase II, TATA box binding protein (TBP)-associated factor	1.9337	3.62E-05	DOWN
Slc7a11 :: solute carrier family 7 (cationic amino acid transporter, y+ system), member 11	7.3157	3.72E-05	DOWN
B4galnt4 :: beta-1,4-N-acetyl-galactosaminyl transferase 4	2.2432	3.81E-05	DOWN
Gls2 :: glutaminase 2 (liver, mitochondrial)	2.3353	3.93E-05	DOWN
Fbln2 :: fibulin 2	2.0405	4.05E-05	DOWN
2610021K21Rik :: RIKEN cDNA 2610021K21 gene	2.9111	4.51E-05	DOWN
Gstcd :: glutathione S-transferase, C-terminal domain containing	1.8478	0.0000465	DOWN
Rps6ka6 :: ribosomal protein S6 kinase polypeptide 6	2.1709	5.28E-05	DOWN
Hspa1l :: heat shock protein 1-like	1.9999	5.89E-05	DOWN
Dyrk3 :: dual-specificity tyrosine-(Y)-phosphorylation regulated kinase 3	2.8598	6.15E-05	DOWN
Hmgn5 :: high-mobility group nucleosome binding domain 5	3.2189	6.36E-05	DOWN
Deptor :: DEP domain containing MTOR-interacting protein	4.1093	6.37E-05	DOWN

Supplementary Table S2. Top 20 gene sets identified from the C5 GO gene sets that are enriched in the Id- knockdown K1 cells.

C5 GO Gene Set	Normalised Enrichment Score	P value	Direction
IMMUNE_RESPONSE	2.136929	<0.0001	UP
G_PROTEIN_COUPLED_RECEPTOR_BINDING	2.1243143	<0.0001	UP
CHEMOKINE_RECEPTOR_BINDING	2.111146	<0.0001	UP
CHEMOKINE_ACTIVITY	2.0914385	<0.0001	UP
I_KAPPAB_KINASE_NF_KAPPAB_CASCADE	2.0558612	<0.0001	UP
DEFENSE_RESPONSE	2.0529644	<0.0001	UP
JAK_STAT_CASCADE	2.0520313	<0.0001	UP
HEMATOPOIETIN_INTERFERON_CLASSD200_DOMAIN_CYTOKINE_RECEPTOR_ACTIVITY	2.0426128	<0.0001	UP
LOCOMOTORY_BEHAVIOR	2.0126882	<0.0001	UP
REGULATION_OF_I_KAPPAB_KINASE_NF_KAPPAB_CASCADE	2.0115595	<0.0001	UP
M_PHASE	-2.5211284	<0.0001	DOWN
CELL_CYCLE_PROCESS	-2.5124967	<0.0001	DOWN
RNA_PROCESSING	-2.4857798	<0.0001	DOWN
CHROMOSOME	-2.3979888	<0.0001	DOWN
M_PHASE_OF_MITOTIC_CELL_CYCLE	-2.3873682	<0.0001	DOWN
MITOSIS	-2.386327	<0.0001	DOWN
CHROMOSOMEPERICENTRIC_REGION	-2.366616	<0.0001	DOWN
SPINDLE	-2.3466449	<0.0001	DOWN
CHROMOSOME_ORGANIZATION_AND_BIOGENESIS	-2.3239574	<0.0001	DOWN
MITOTIC_CELL_CYCLE	-2.3222191	<0.0001	DOWN

Supplementary Table S3. Top 20 gene sets identified from the C6 Oncogenic Signatures that are enriched in the Id- knockdown K1 cells

C6 Oncogenic Gene Set	Normalised Enrichment Score	P value	Direction
LTE2_UP.V1_DN	2.389604	<0.0001	UP
MEK_UP.V1_DN	2.244798	<0.0001	UP
VEGF_A_UP.V1_UP	2.124735	<0.0001	UP
WNT_UP.V1_DN	2.070847	<0.0001	UP
PKCA_DN.V1_UP	1.969476	<0.0001	UP
MYC_UP.V1_DN	1.865835	<0.0001	UP
BMI1_DN_MEL18_DN.V1_DN	1.860512	<0.0001	UP
MEL18_DN.V1_DN	1.845022	<0.0001	UP
BMI1_DN.V1_DN	1.83551	<0.0001	UP
STK33_UP	1.819187	<0.0001	UP
RPS14_DN.V1_DN	-2.3749187	<0.0001	DOWN
HOXA9_DN.V1_DN	-2.214685	<0.0001	DOWN
CSR_LATE_UP.V1_UP	-2.1530662	<0.0001	DOWN
PRC2_EZH2_UP.V1_UP	-2.0872	<0.0001	DOWN
VEGF_A_UP.V1_DN	-2.0221975	<0.0001	DOWN
RB_P107_DN.V1_UP	-2.0133445	<0.0001	DOWN
E2F1_UP.V1_UP	-2.0126765	<0.0001	DOWN
MYC_UP.V1_UP	-1.8403969	<0.0001	DOWN
STK33_DN	-1.8023152	<0.0001	DOWN
NFE2L2.V2	-1.7664328	<0.0001	DOWN

Supplementary Table S4. Top 20 gene sets identified from the C2 curated gene sets that are enriched in the Id-knockdown K1 cells.

C2 Curated Gene Set	Normalised Enrichment Score	P value	Direction
BROWNE_INTERFERON_RESPONSIVE_GENES	2.893714	<0.0001	UP
TAKEDA_TARGETS_OF_NUP98_HOXA9_FUSION_3D_UP	2.889862	<0.0001	UP
ICHIBA_GRAFT_VERSUS_HOST_DISEASE_D7_UP	2.805356	<0.0001	UP
SANA_TNF_SIGNALING_UP	2.760641	<0.0001	UP
SANA_RESPONSE_TO_IFNG_UP	2.652481	<0.0001	UP
DER_IFN_ALPHA_RESPONSE_UP	2.650679	<0.0001	UP
DAUER_STAT3_TARGETS_DN	2.568591	<0.0001	UP
DER_IFN_BETA_RESPONSE_UP	2.544266	<0.0001	UP
MOSERLE_IFNA_RESPONSE	2.528648	<0.0001	UP
RADAEVA_RESPONSE_TO_IFNA1_UP	2.456697	<0.0001	UP
ROSTY_CERVICAL_CANCER_PROLIFERATION_CLUSTER	-3.2496483	<0.0001	DOWN
SOTIRIOU_BREAST_CANCER_GRADE_1_VS_3_UP	-3.1307108	<0.0001	DOWN
WONG_EMBRYONIC_STEM_CELL_CORE	-3.0442135	<0.0001	DOWN
GRAHAM_CML_DIVIDING_VS_NORMAL_QUIESCENT_UP	-2.9951138	<0.0001	DOWN
KOBAYASHI_EGFR_SIGNALING_24HR_DN	-2.950584	<0.0001	DOWN
LEE_EARLY_T_LYMPHOCYTE_UP	-2.9474769	<0.0001	DOWN
PUJANA_BRCA2_PCC_NETWORK	-2.91798	<0.0001	DOWN
LI_WILMS_TUMOR_VS_FETAL_KIDNEY_1_DN	-2.8696544	<0.0001	DOWN
SHEDDEN_LUNG_CANCER_POOR_SURVIVAL_A6	-2.8691382	<0.0001	DOWN
FURUKAWA_DUSP6_TARGETS_PCI35_DN	-2.841147	<0.0001	DOWN

Supplementary Table S5. List of differentially expressed Myc co-factors in Id + Robo1 KD in comparison to Id1/3 KD alone obtained from the RNA-Seq data. Red indicates negative regulators and green indicates positive regulators.

**Downregulated by Robo1 KD in the absence of Id1/3**	**Upregulated by Robo1 KD in the absence of Id1/3**	**Unchanged by Robo1 KD in the absence of Id1/3**
Bptf	Nme2	Ash2l
Pim1	MlxIP	Bptf
Rlim	Mlx	Brd4
Zbtb17	Actl6a	Cdk8
Smad3	Kat2a	Cdk9
Hbp1	Ruvbl2	Dot1l
Mxd4	Skp2	Ep300
Tsc22d1	Banf1	Ep400
Hdac2	Ash2l	Kat5
Mycl	Atad2	Kdm5a
Rnf115	Ccnt1	Max
	Npm1	Med26
	Aurka	Myc
	Clock	Mycn
	Mxd3	Sirt1
	Hdac1	Smarcb1
	Suds3	Snip1
	Sap30	Trrap
	Sap18	Arntl
	Rbbp7	Fbxw7
	Fbxw7	Huwe1
	Trpc4ap	Mga
	Bin1	Mnt
	Rbl1	Mxd1
		Rpl11
		Sin3a
		Sin3b
		Btg1

Supplementary Table S6. The list and the concentrations of antibodies used for western blotting in the study.

Antibody	Manufacturer	Catalogue #	Concentration
Rabbit anti-mouse Id1 (primary)	BioCheck (Burlingame, CA, USA)	BCH-1/37-2	1:500
Rabbit anti-human/mouse Id3 (primary)	BioCheck (Burlingame, CA, USA)	BCH-4/#17-3	1:500
Mouse anti- β-actin (primary)	AbCam (Cambridge, UK)	Ab6276	1:4000
Horse anti-mouse IgG-HRP (secondary)	Cell Signaling (Beverly, MA, USA)	7054	1:8000
Goat anti-rabbit IgG-HRP (secondary)	Cell Signaling (Beverly, MA, USA)	7054	1:5000

Supplementary Table S7. The list and the concentrations of antibodies used for Immunohistochemistry in the study.

Antibody	Manufacturer	Catalogue #	Concentration
Rabbit anti-mouse Id1 (primary)	BioCheck (Burlingame, CA, USA)	BCH-1/37-2	1:50
Rabbit anti-human/mouse Id3 (primary)	BioCheck (Burlingame, CA, USA)	BCH-4/#17-3	1:50
Rabbit anti-human Id1 (primary)	BioCheck (Burlingame, CA, USA)	BCH-1/195-14	1:50
Rabbit anti-human/mouse CK14 (Primary)	Covance (Princeton, NJ, USA)	PRB-155P	1:8000
Rabbit anti-human/mouse CK8 (Primary)	Cell Signaling (Beverly, MA, USA)	7054	1:5000
