# Supplementary figures and images for: Id Proteins Promote a Cancer Stem Cell Phenotype in Mouse Models of Triple Negative Breast Cancer via Negative Regulation of Robo1
Source: Front Cell Dev Biol. 2020 Jul 17;8:552. doi: 10.3389/fcell.2020.00552 (PMC7380117; doi:10.3389/fcell.2020.00552)

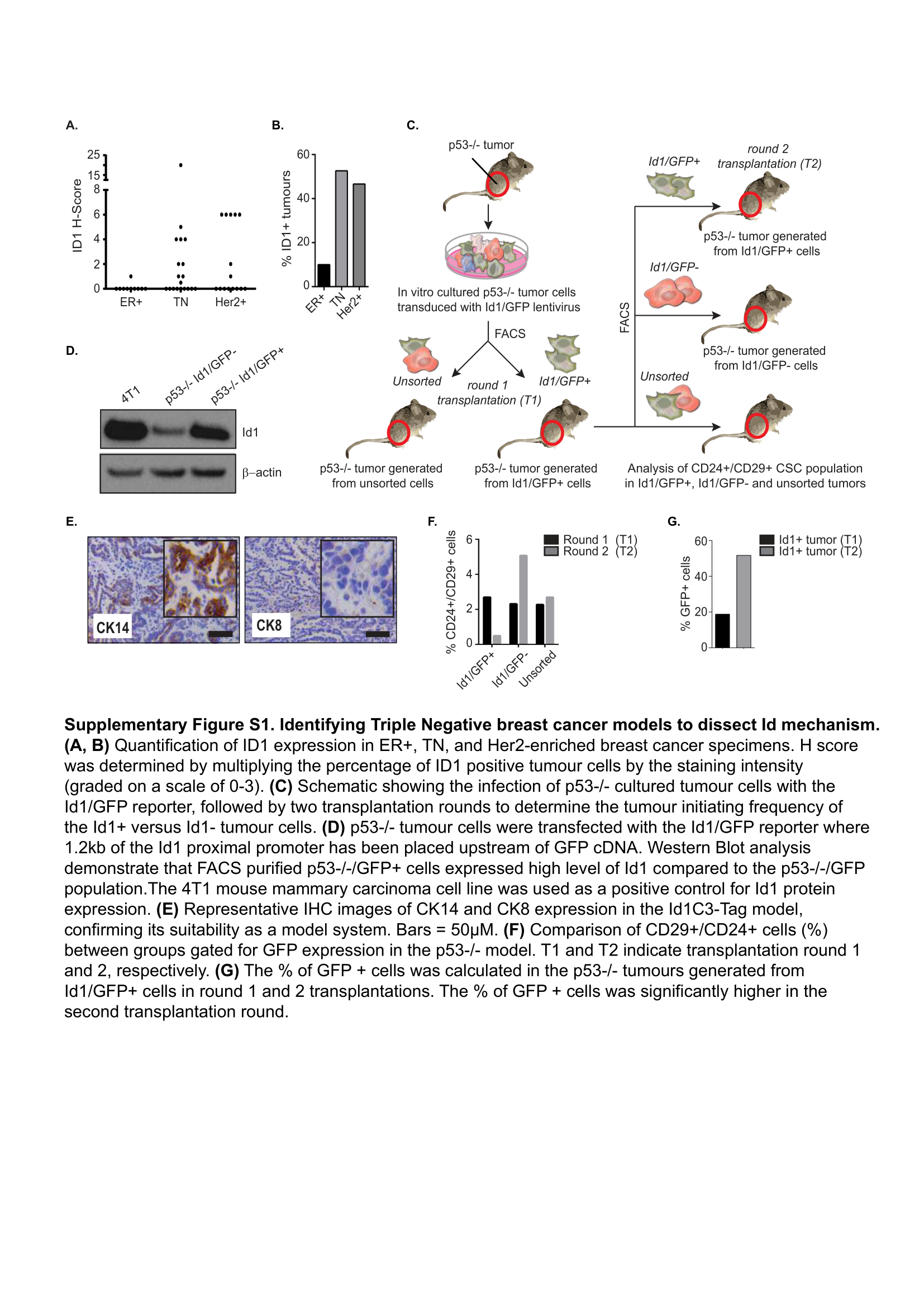

Supplement: Supplementary file 2 [file Image_1.TIFF]

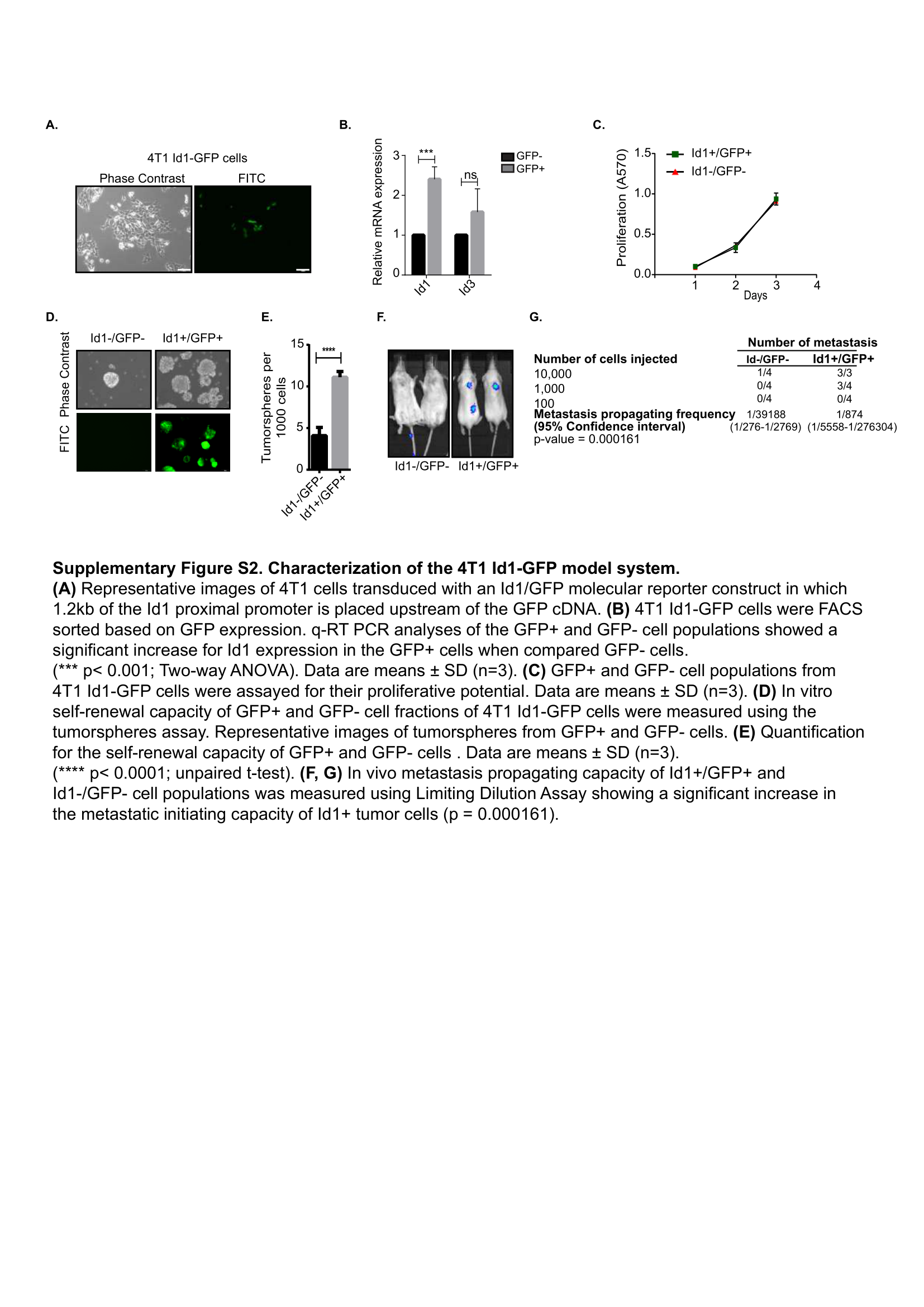

Supplement: Supplementary file 3 [file Image_2.TIFF]

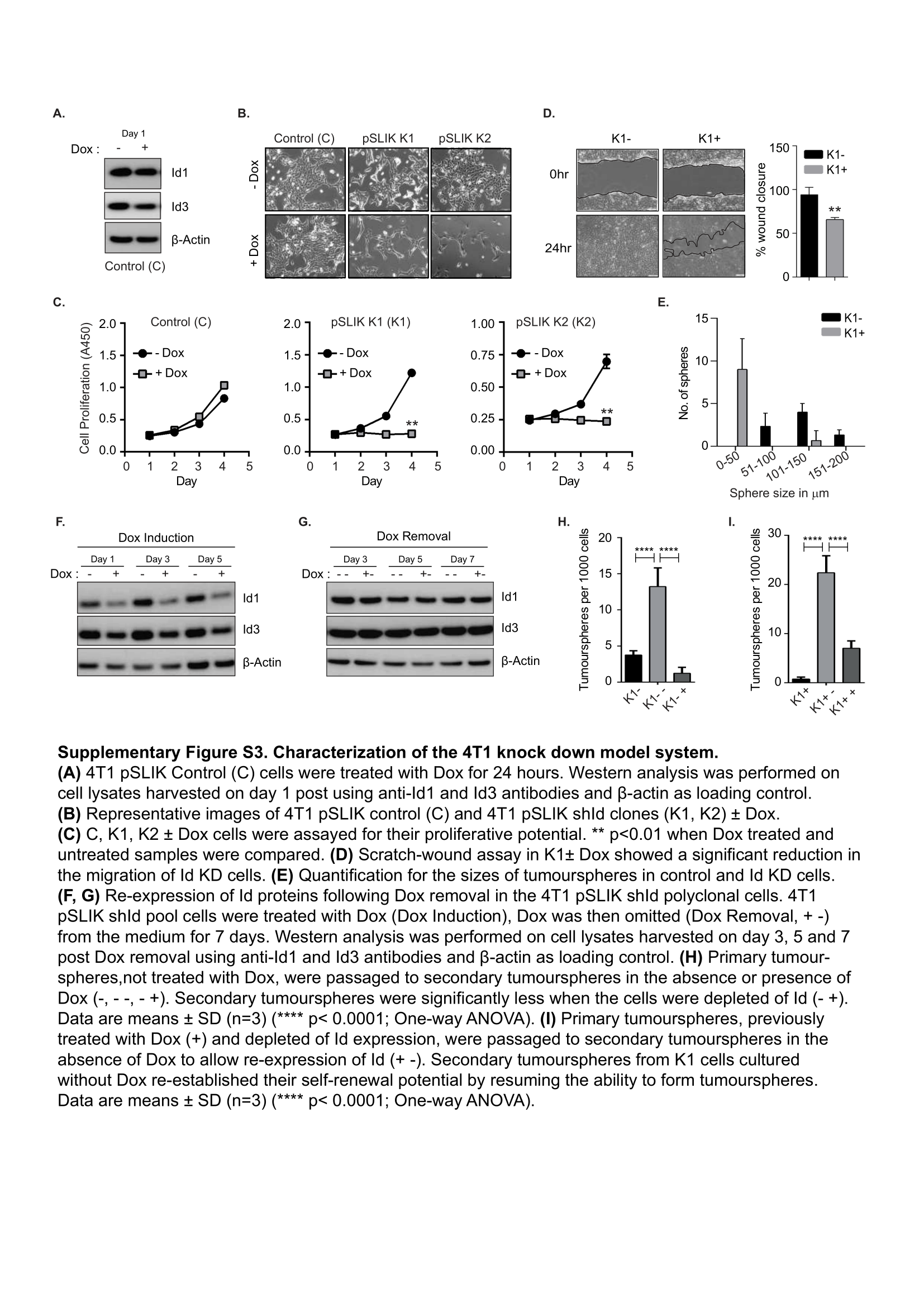

Supplement: Supplementary file 4 [file Image_3.TIFF]

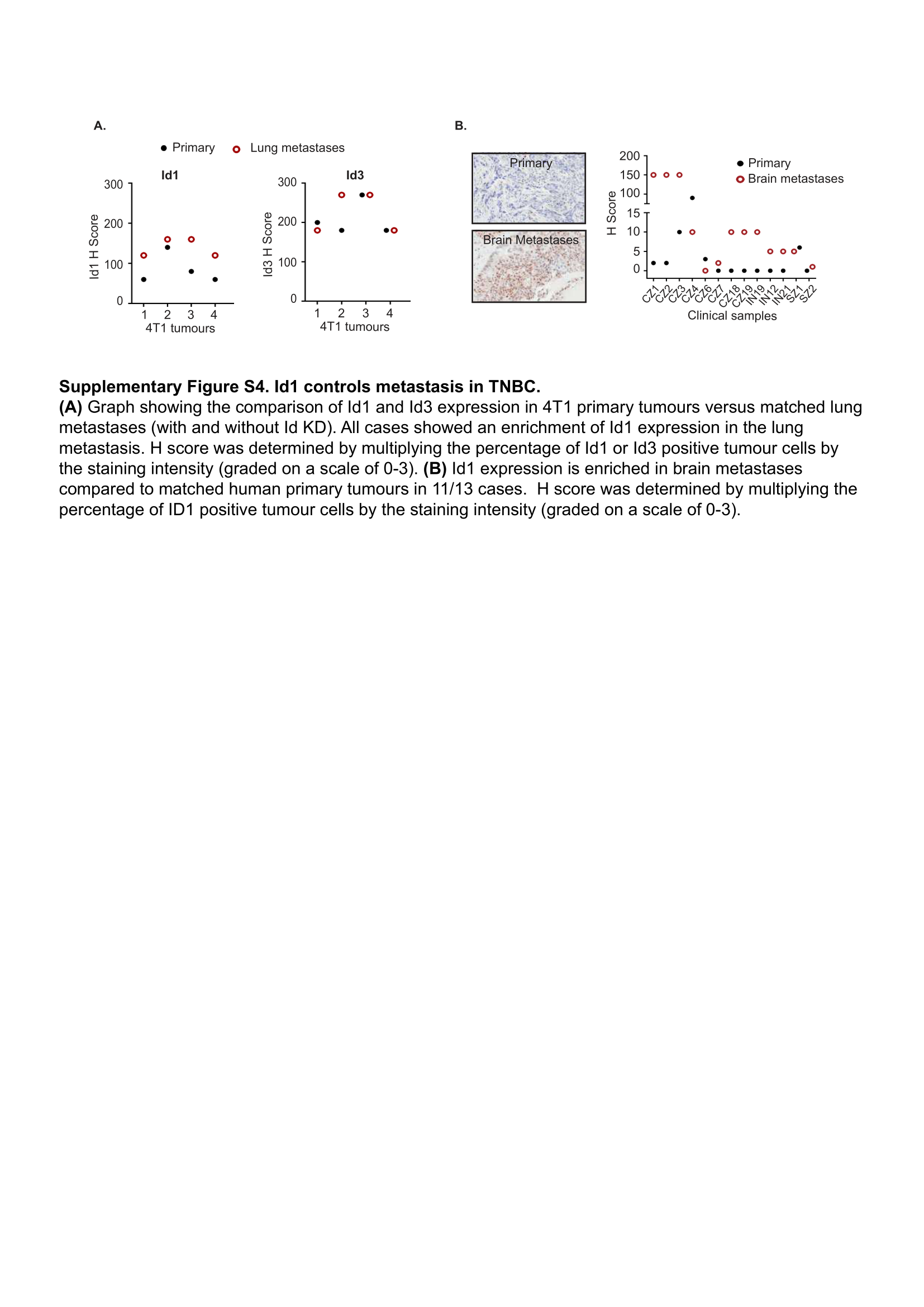

Supplement: Supplementary file 5 [file Image_4.TIFF]

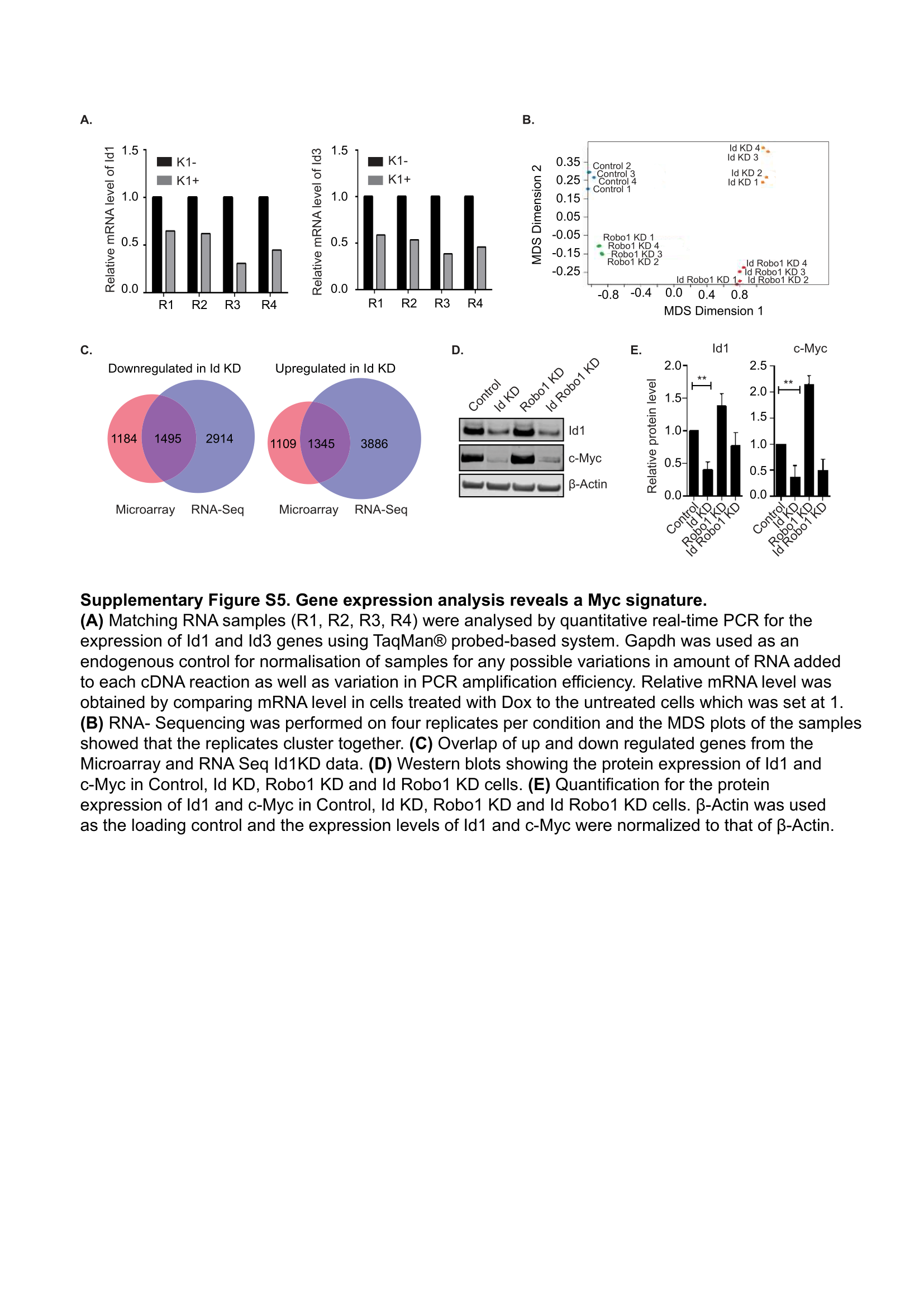

Supplement: Supplementary file 6 [file Image_5.TIFF]
